# Effects of DJK-5 and chlorhexidine on exopolysaccharide volume and pH in oral biofilms

**DOI:** 10.1186/s12903-023-03381-5

**Published:** 2023-09-30

**Authors:** Binwen Chen, He Liu, Zhejun Wang, Jingzhi Ma, Ya Shen

**Affiliations:** 1 Department of Stomatology, Tongji Hospital, Tongji Medical College, Huazhong University of Science and Technology, Wuhan, 430030 Hubei Province China; 2 Department of Oral Biological & Medical Sciences, Faculty of Dentistry, University of British Columbia, Vancouver, Canada

**Keywords:** Chlorhexidine, Confocal laser scanning microscope, DJK-5, Exopolysaccharides, Oral biofilms, pH

## Abstract

**Background:**

Exopolysaccharides (EPS) are essential constituents of the extracellular matrix within oral biofilms and are significantly influenced by the local microenvironment. This study aimed to investigate the impact of two distinct antimicrobial agents, DJK-5 and chlorhexidine (CHX), on the EPS volume and pH levels in oral biofilms.

**Methods:**

Oral biofilms obtained from two donors were cultured on hydroxyapatite discs for durations of 3 days, 1 week, 2 weeks, 3 weeks, and 4 weeks. Subsequently, these biofilms were subjected to treatment with 10 µg/mL DJK-5 or 2% CHX for 3 min. The impact of these antimicrobial treatments on factors such as the proportion of dead bacterial, in situ pH, and EPS volume within the biofilms was assessed using corresponding fluorescent probes. The examination was carried out utilizing confocal laser scanning microscopy, and the resulting images were analyzed with a focus on the upper and lower layers of the biofilm, respectively.

**Results:**

DJK-5 exhibited a more potent bactericidal effect compared to CHX across the 3-day to 4-week duration of the biofilm (*P* < 0.05). The biofilms were acidic, with the upper layer being less acidic than the lower layer (*P* < 0.05). Both antimicrobial agents increased the pH, but DJK-5 had a greater effect than CHX (*P* < 0.05). The volume of EPS was significantly lower in DJK-5 treated biofilms compared to that of CHX, regardless of age or layer (*P* < 0.05).

**Conclusion:**

DJK-5 exhibited superior effectiveness in reducing viable bacteria and EPS volume, as well as in raising extracellular pH, as compared to chlorhexidine.

## Background

A biofilm is typically composed of a matrix produced by bacteria, which envelopes and establishes a multicellular structure along with the bacterial cells [1]. This matrix comprises various substances including exopolysaccharides (EPS), proteins, lipids, nucleic acids, and lipoteichoic acids [2–4]. Among these components, EPS have garnered significant attention due to their vital role as essential constituents of the biofilm matrix and their categorization as virulence factors in caries development [5]. Prior research efforts focusing on EPS have primarily centered around cariogenic biofilms of *Streptococcus mutans* mono-species [6, 7], laboratory-constructed biofilms consisting of a limited number of species [8], or polymicrobial biofilms derived from saliva samples [9]. Furthermore, investigations into the pH of the biofilm matrix have gone hand in hand with EPS research, as a reduction in pH can cultivate a more favourable microenvironment for acid-tolerant bacteria.

The composition of the biofilm matrix can be influenced by various factors, including the bacterial species, local microenvironment, and the duration of biofilm growth [10]. Notably, Koo et al. found that *S. mutans* and/or its metabolic products played a pivotal role in the formation of the biofilm matrix. When *Streptococcus oralis* and *Actinomyces naeslundii* were cultured together without *S. mutans*, neither matrix formation nor EPS-enmeshed microcolonies emerged [11]. Moreover, Xiao et al. observed that employing a 1% sucrose solution could lead to an increase in biofilm biomass. Biofilms cultivated with 1% sucrose displayed greater thickness and contained a tenfold increment in both EPS and bacterial biomass when compared to those grown with a 1% glucose. Interestingly, bacterial cells that were not organized into EPS-microcolonies exhibited higher susceptibility to chlorhexidine (CHX) treatment, although the impact of CHX on EPS was not specifically examined in that study [8]. Another prior study also highlighted that the combination of oleic acid with fluoride significantly hindered EPS formation [6].

CHX is a prevalent antimicrobial agent in dental care, available in concentrations ranging from 0.12 to 2%. Notably, the most commonly employed concentration of CHX for endodontic treatment is 2%. Its cationic charge enables it to bind to negatively charged bacterial surfaces, thereby damaging the cell wall. However, the effectiveness of CHX has been found to diminish in mature biofilms, probably due to factors such as the presence of EPS, the nutritionally deprived physiological state of biofilm bacteria, and the presence of “persisters” [12, 13].

**Fig. 1:**
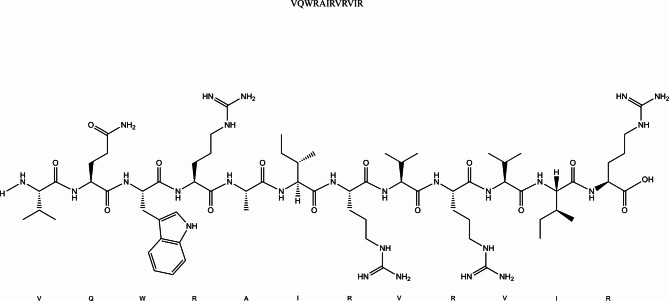
Amino acid structure of DJK-5

Recently, a novel category of antimicrobial peptides has been developed for antibiofilm purposes, with DJK-5 being among the notable representatives. DJK-5 is a D-enantiomeric cationic peptide composed of twelve amino acid residues, and its amino acid sequence is VQWRAIRVRVIR (Fig. 1). Research has demonstrated that DJK-5 effectively eradicates oral biofilms in both young and mature stages [14]. One distinctive attribute of CHX is its prolonged impact. Research on DJK-5 has indicated that it exhibits a persistent bactericidal effect similar to CHX for up to 7 days [15]. Furthermore, the period required for biofilm regrowth subsequent to DJK-5 exposure was observed to be longer compared to that following exposure to CHX [16].

Conventional approaches for investigating EPS and pH in biofilms typically involve assessing the dry weight of polysaccharides [17] or employing in-dwelling or micro-touch electrodes [18]. However, these methods can potentially disrupt the biofilm’s microenvironment. Fortunately, fluorescent dyes have been developed more recently, enabling the detection and visualization of the three-dimensional arrangement of the biofilm as well as the distribution of pH within it [19, 20]. The goal of this study was to construct a model representing the three-dimensional structure of the EPS matrix and pH distribution within oral biofilms, utilizing specialized fluorescent dyes for EPS staining, pH detection, and confocal laser scanning microscope (CLSM). Additionally, the study aimed to assess the impact of two distinct antimicrobial agents (CHX and DJK-5) on the EPS volume and pH of oral biofilms. The null hypothesis proposed that antimicrobial treatment would not affect the EPS volume and pH of oral biofilms.

## Methods

### Peptide synthesis

The peptide DJK-5 was synthesized by GenScript (Piscataway, NJ, USA) using solid-phase 9-fluorenylmethoxy carbonyl chemistry, following the protocol outlined in a previous study [21]. The peptide was subsequently purified to a minimum purity of 95% using reverse-phase high-performance liquid chromatography, and its identity was confirmed by amino acid analysis. To create a peptide stock solution, the peptide powder was suspended in deionized water to achieve a concentration of 100 µg/mL.

### Biofilm model

To cultivate oral biofilms, sterilized collagen-coated hydroxyapatite (HA) discs (Clarkson Chromatography Products, PA, USA) were utilized. In each well of a 24-well plate, a single disc was placed. A solution of bovine dermal type I collagen (10 mg/mL collagen in 0.012 N HCl in deionized water) (Cohesion, CA, USA) was introduced into each well to cover the disc surfaces, after which the plate was left overnight at 4 °C.

Subgingival plaque was collected from two healthy adult volunteers using wooden toothpicks and was suspended in brain-heart infusion (BHI) broth (Becton Dickinson, MD, USA). The optical density of the dispersed plaque suspension was adjusted to 0.1 at 405 nm using a microplate reader (ELx808 Absorbance Reader; BioTek Instruments, Inc., VT, USA). Subsequently, the suspension was diluted tenfold with fresh BHI. Following the removal of the coating collagen solution, 2 mL of the diluted plaque suspension was added to each well. The plates were then anaerobically incubated at 37 °C for periods of 3 days, 1 week, 2 weeks, 3 weeks, and 4 weeks, with the BHI broth being renewed every week.

### Treating biofilm with antimicrobial agents

Following each incubation period, the biofilm-covered discs were subjected to a 3-minute treatment with either 10 µg/mL DJK-5 or 2% CHX. For each treatment, 50 µL of the respective irrigant was applied to cover the surface of them. Subsequently, the discs were rinsed using physiological saline. As a negative control, biofilms treated with deionized water for 3 min were utilized. Three HA discs were assigned to each group.

### Bacterial viability measurement and visualisation

After being exposed to the aforementioned solutions, the specimens were stained using a bacterial viability fluorescent probe (LIVE/DEAD Baclight Kit; ThermoFisher Scientific, MA, USA). The staining was then examined through CLSM, following the procedures detailed in previous studies [22, 23]. Dead bacteria were stained red, while live bacteria were stained green. For each HA disc, five distinct scanned areas were selected. Subsequently, three-dimensional volume stacks were constructed using Imaris 7.2 software (Bitplane Inc, MN, USA). The total volume of red and green fluorescence was measured, and based on this, the proportion of dead bacteria was calculated. The calculation of the proportion of dead bacteria was performed separately for the upper and lower halves of the biofilm.

### Non-invasive in situ pH measurement and visualisation

In this study, a fluorescent pH indicator, Lysosensor yellow/blue (ThermoFisher Scientific, MA, USA) was employed to determine the in-situ pH within the biofilms. This pH indicator was conjugated to dextran (10 kDa, MW) (Lysosensor yellow/blue Dextran, labelled as “Dextran”), and this conjugated dextran could be incorporated into the synthesis of EPS during the formation of the biofilm matrix [24]. The quantification of pH levels was accomplished by utilizing the fluorescence intensity ratios of the dual-wavelength fluorophore [25].

The fluorescence emission of Lysosensor yellow/blue presents spectral peaks at two distinct wavelengths, namely 452 and 521 nm. These emission spectra exhibit alterations upon protonation, resulting in an increase in fluorescence intensity at 521 nm and a simultaneous decrease at 452 nm as the pH decreases [20]. Consequently, the ratio of fluorescent intensity at these two wavelengths (I450/I520) corresponds to specific pH values. A titration curve, depicting the ratio versus pH (ranging from 3.5 to 7.0) was constructed for both pH probes, as described in a previous study [8]. This curve was then utilized to convert the fluorescence intensity ratios into corresponding pH values.

To conduct Dextran staining, 4 µL of a 0.5 mM working solution of Dextran was added to the 2 mL bacterial suspension in each well to achieve a dye concentration of 1 µM at the beginning of biofilm culturing. Subsequently, an additional 4 µL of the solution was supplemented each week, coinciding with the changing of the BHI broth. Following the staining procedure, the biofilm-covered discs were removed from the BHI broth and then subjected to treatment with the specified antimicrobial agents mentioned earlier, before being prepared for CLSM analysis (FV10i-LIV, Olympus, Canada).

CLSM images were captured. For each disc, five random areas were chosen and then reconstructed using Imaris 7.2 software. Subsequently, the ratios of fluorescent intensity at both wavelengths (I450/I520) were computed to determine the pH values of the biofilm using the previously established titration curve. This procedure was also applied to calculate the pH values for both the upper and lower halves of the biofilm.

### EPS volume measurement and visualisation

A fluorescent marker was incorporated into the EPS during its synthesis to enable visualization of the intact biofilms in three-dimensions [26]. Dextran with a molecular weight of 10 kDa was conjugated to Alexa Fluor 647 (ThermoFisher Scientific, MA, USA), a fluorescent probe with an absorbance/emission peak at 650/668 nm. At the beginning of biofilm development and during its progression, 1 mM Alexa Fluor 647-labelled dextran was incorporated into the BHI broth to facilitate EPS visualization.

For labeling live bacteria, SYTO 9 green-fluorescent nucleic acid stain (485/498 nm; ThermoFisher Scientific, MA, USA) was employed after the application of antimicrobial agents. Following staining, the specimens underwent a 1-minute rinse with physiological saline. The subsequent acquisition and reconstruction of CLSM images were carried out using the methods detailed earlier. The volume of EPS and live bacteria was quantified, allowing for the calculation of volume ratios pertaining to live bacteria to EPS. These calculations encompassed both the volume and ratio of the upper and lower halves of the biofilm.

### Statistical analysis

The statistical analysis was performed using SPSS for Mac, Version 25 (IBM Corp, NY, USA). One-way analysis of variance was employed, followed by the application of the Tukey post hoc multiple comparison test when applicable. The statistical significance level was set at *P* < 0.05.

## Results

### Bactericidal effect of DJK-5 and CHX

**Fig. 2:**
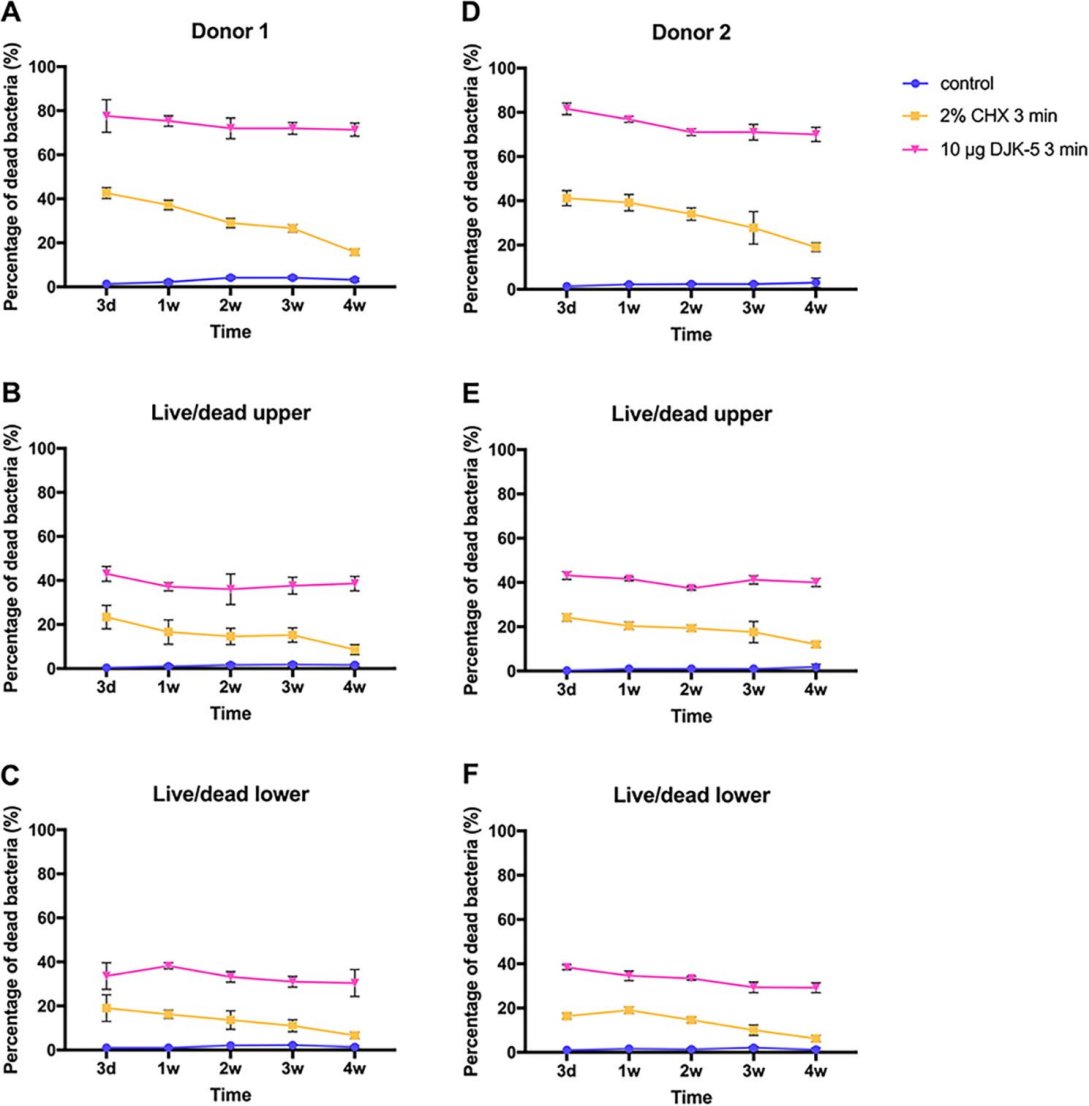
The proportion of dead bacterial within biofilms of different ages from both donors, treated with different antimicrobial agents. (**A**, **B**, **C**: donor 1; **D**, **E**, **F**: donor 2; **A** and **D**: whole biofilm; **B** and **E**: upper layer of the biofilm; **C** and **F**: lower layer of the biofilm)

DJK-5 displayed a more potent bactericidal effect than CHX within the 3-day to 4-week duration in the biofilm (*P* < 0.05). Furthermore, in the DJK-5 treated group, the proportion of dead bacteria reached a steady range of 80 − 70% within both young and mature biofilms. Conversely, in the CHX-treated group, the percentage of dead bacteria exhibited a notable decline, dropping from 40% in young biofilms to 16% in mature biofilms. Within the control group, the age of the biofilm had no impact on the proportion of dead bacteria, which consistently remained below 10% (Fig. 2).

**Fig. 3:**
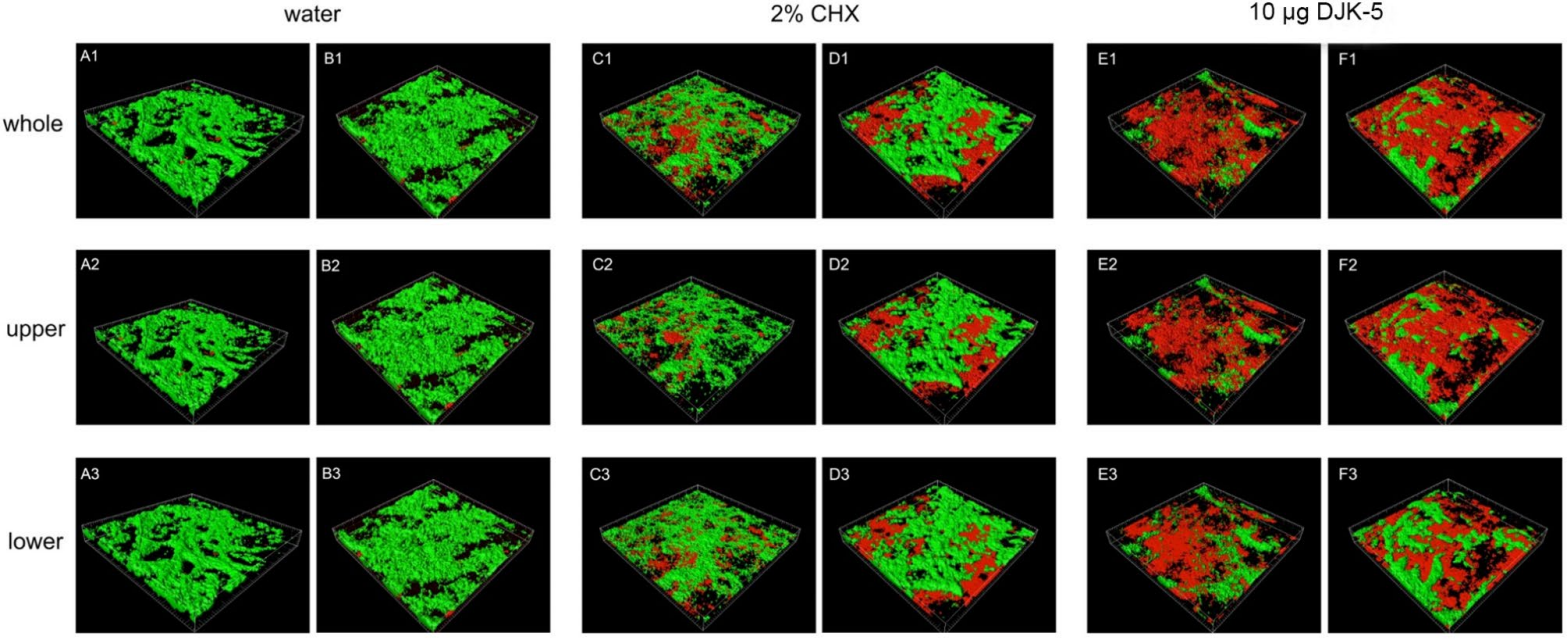
Representative CLSM images of live and dead bacteria within 3-day and 3-week biofilms that underwent treatment with different antimicrobial agents. (**A**, **C**, and **E**: 3-day; **B**, **D** and **F**: 3-week; Green: live bacteria; Red: dead bacteria)

Upon dividing the biofilm into upper and lower layers and subjecting them to separate analyses, it was observed that the upper layer of the biofilm treated with both DJK-5 and CHX exhibited a higher proportion of dead bacteria than the lower layer, regardless of the age of the biofilm (*P* < 0.05) (Fig. 3). In the lower layer of the 4-week-old biofilm subjected to CHX treatment, the proportion of dead bacteria was found to be comparable to that of the control group (*P* > 0.05) (Fig. 2C and F).

### pH distribution in time and space of the antimicrobial agents-treated and untreated biofilms

**Fig. 4:**
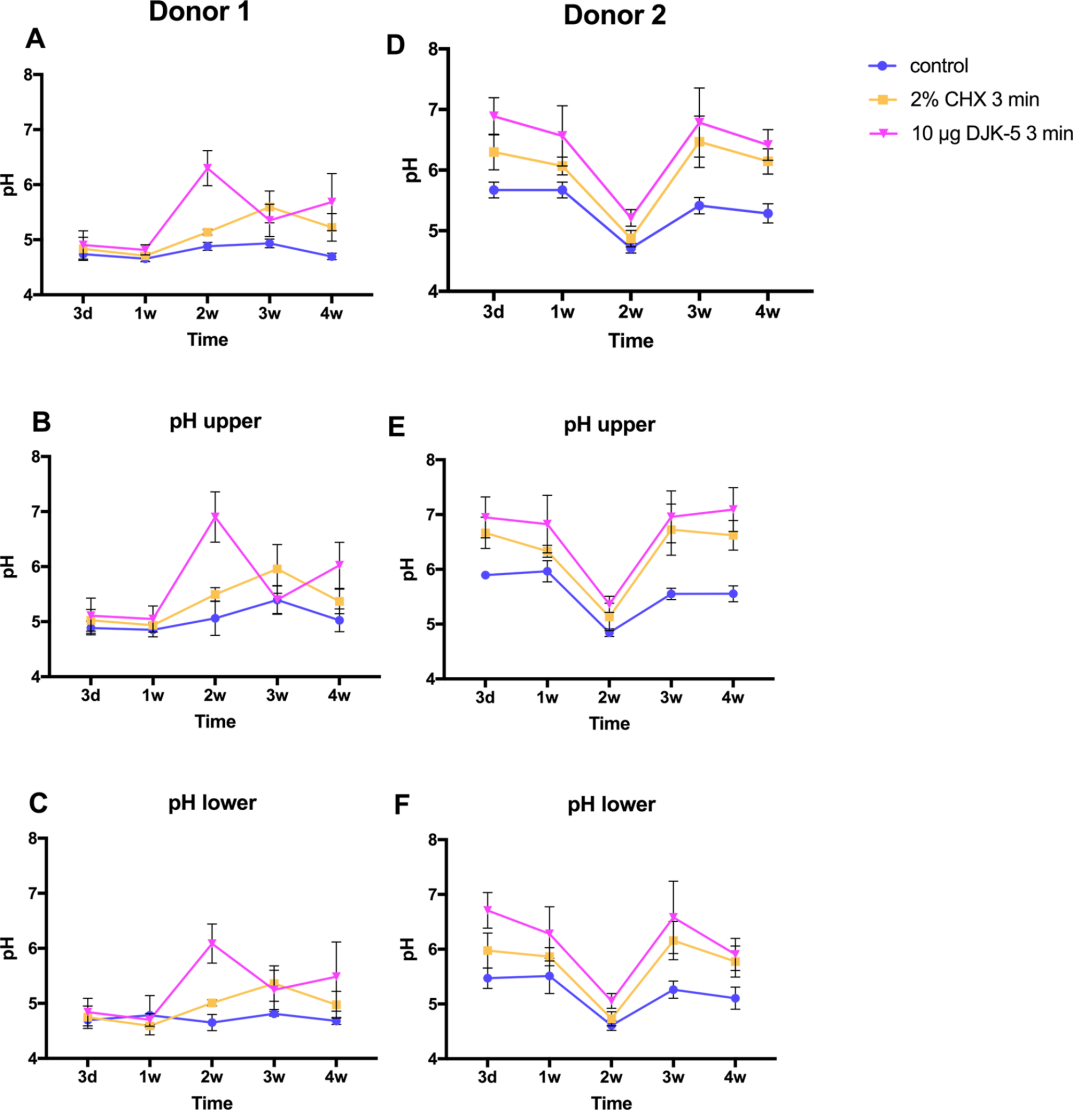
The pH levels of biofilms of different ages from the two donors, treated with different antimicrobial agents. (**A**, **B**, **C**: donor 1; **D**, **E**, **F**: donor 2; **A** and **D**: whole biofilm; **B** and **E**: upper layer of the biofilm; **C** and **F**: lower layer of the biofilm)

The untreated biofilm from both donors exhibited acidity, with extracellular pH ranging from 4.67 to 4.93 for donor 1 and from 4.71 to 5.67 for donor 2 (Fig. 4A and D). The pH trend over time in the biofilms differed between the two donors, both treated and untreated by antimicrobial agents. For donor 1, the pH was slightly lower in the younger biofilms (3-day and 1-week) and tended to stabilize when mature (2 weeks and beyond). Conversely, for donor 2, no significant difference was observed in pH in the biofilms over time, except for a significantly lower pH in the 2-week biofilm (*P* < 0.05).

The antimicrobial agents were capable of increasing pH in the biofilms, although it remained acidic. DJK-5 could raise the pH to almost neutral (6.88) in the 3-day biofilm of donor 2 (Fig. 4D). In both donors, the DJK-5 treated group resulted in a higher pH than the CHX-treated group, with significant differences observed in all biofilm ages of donor 1 (*P* < 0.05) (Fig. 4A, B, and C). This difference was observed only in the young biofilm (3-day and 1-week) of donor 2 (Fig. 4D, E, and F) (*P* < 0.05). The pH in the upper layer (Fig. 4B and E) was higher than that in the lower layer (Fig. 4C and F) for all treatment groups and time periods in both donors.

### Growth of oral biofilm exopolysaccharides over time and the effect of antimicrobials on exopolysaccharides

**Fig. 5:**
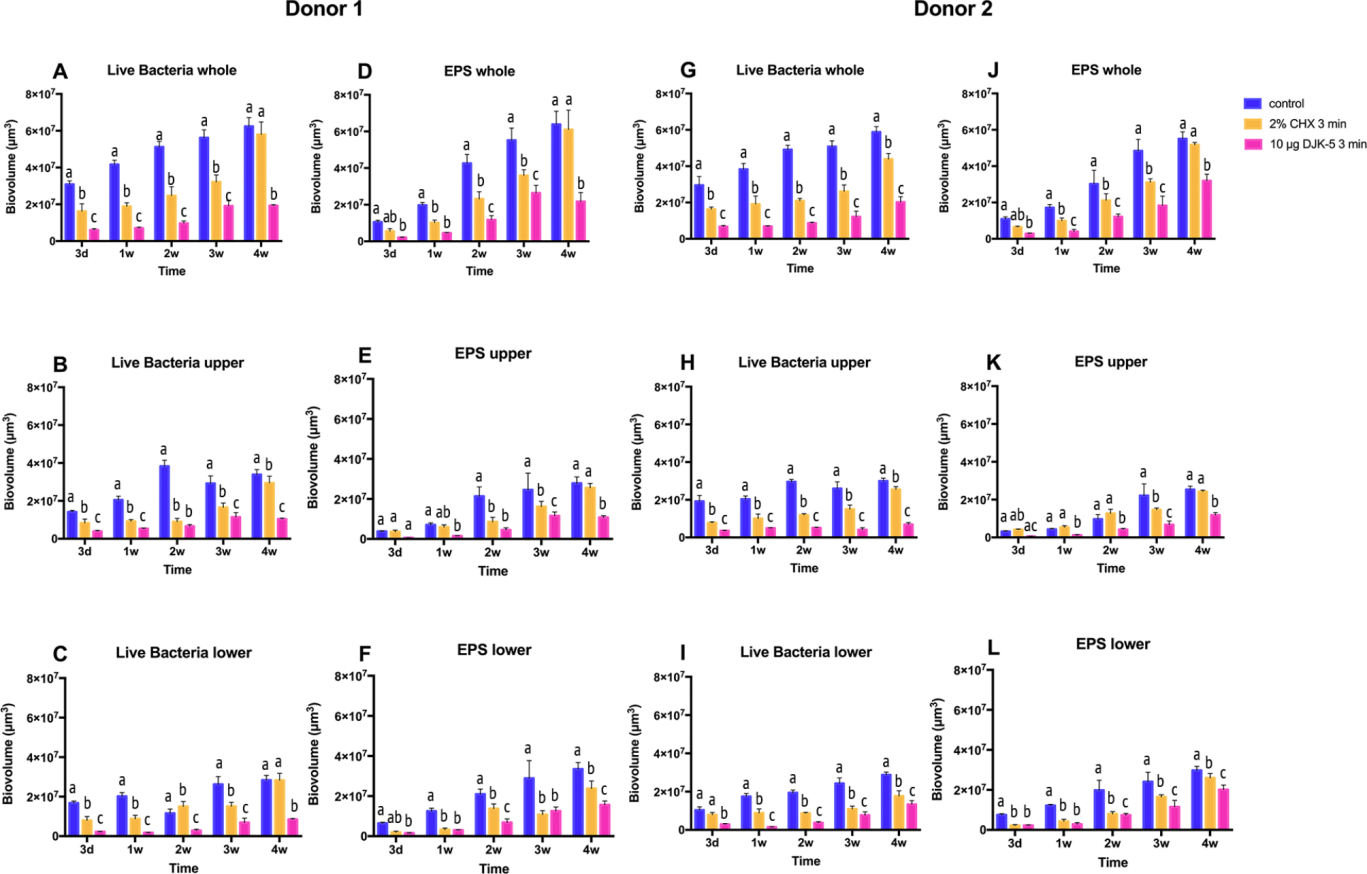
The biovolume of EPS and live bacteria in biofilms of different ages from both donors, subject to different antimicrobial treatments. (**A**, **D**, **G** and **J**: whole biofilm; **B**, **E**, **H** and **K**: upper layer; **C**, **F**, **I** and **L**: lower layer). Different lowercase letters represent significant differences within the group (*P* < 0.05)

**Fig. 6:**
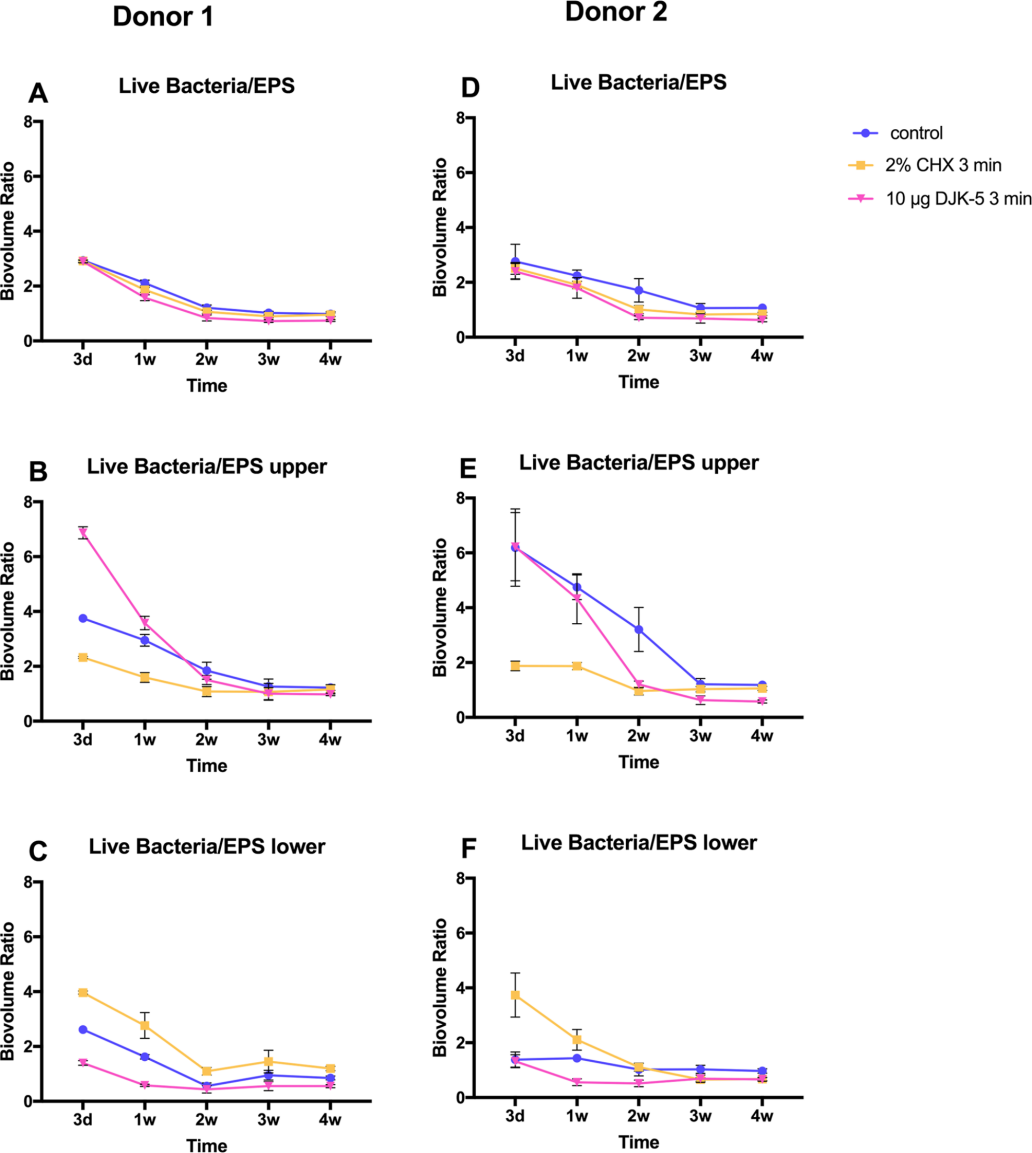
The ratio of live bacteria to EPS within biofilms of different ages from both donors, treated with different antimicrobial agents. (**A** and **D**: whole biofilm; **B** and **E**: upper layer of biofilm; **C** and **F**: lower layer of biofilm)

The volume of EPS increased with time for both donors (Fig. 5D and J), as did the number of live bacteria (Fig. 5A and G). In young biofilms, the volume of live bacteria exceeded that of EPS, but the volume of EPS increased rapidly from 1 to 2 weeks of culturing. The volume ratio of live bacteria to EPS reached approximately 1:1 and remained stable as the biofilm matured (Fig. 6A and D).

The impact of antimicrobial agents on EPS volume depended on the age of the biofilm. In the 3-day biofilm, no differences in EPS volume were observed among the three treatment groups. However, in the 1-week to 3-week biofilms, both DJK-5 and CHX significantly reduced EPS volume, with DJK-5 causing a greater reduction compared to CHX. In the 4-week biofilm, the CHX group did not show a significant decrease in EPS volume, while the DJK-5 group still exhibited a significant reduction (Fig. 5D and J). The live bacteria/EPS ratio was slightly decreased by CHX and DJK-5 in biofilms aged 1 week or older. This suggests that CHX and DJK-5 eliminated a relatively higher number of live bacterial cells compared to EPS. However, in the 3-day biofilm, the ratio remained unchanged by the antimicrobial agents.

EPS and live bacterial volume were also separately calculated for the upper (Fig. 5B, E, H, and K) and lower layers (Fig. 5C, F, I, and L) of the biofilms. In the control and DJK-5 treated groups, a higher EPS volume was observed in the lower layer, while in the CHX group, no significant difference in EPS volume was found between the lower and upper layers (Fig. 5E, F, K, and L). Furthermore, the live bacteria/EPS ratio of the CHX-treated group was smaller than that of the control group in the upper layer and larger than that in the control group in the lower layer. This suggests that the volume of EPS was relatively increased in the upper layer of the biofilm after CHX treatment (Fig. 6B and C). An exception was observed in mature biofilms (3 weeks and 4 weeks) of donor 2 treated with CHX; the live bacteria/EPS remained unchanged in both the upper and lower layers (Fig. 6E and F).

**Fig. 7:**
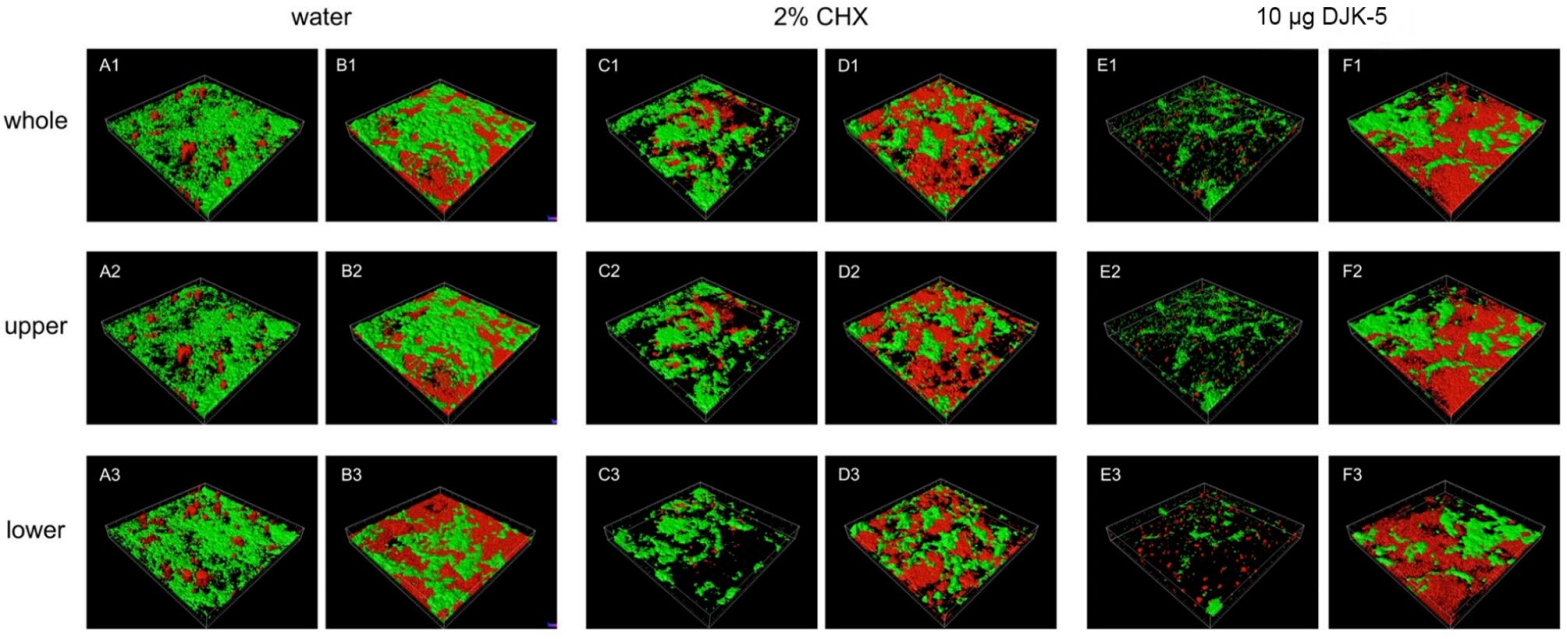
Representative CLSM images of live bacteria and EPS within 3-day and 3-week biofilms treated with different antimicrobial agents. (**A**, **C** and **E**: 3-day; **B**, **D** and **F**: 3-week; Green: live bacteria; Red: EPS)

DJK-5 also had distinct effects on the composition of live bacteria and EPS in the upper and lower layers of the two donors. In the upper layer of the 3-day and 1-week biofilms of donor 1, the live bacteria/EPS ratio was exceptionally large, probably because EPS was almost entirely removed by DJK-5 (Fig. 6B). This ratio was smaller than that of the control in the lower layer, indicating that more live bacteria were reduced than EPS (Fig. 6C). In the young biofilm of donor 2, the live bacteria/EPS ratio of the DJK-5 treated group was similar to that of the control in both the upper and lower layers, showing that live bacteria and DJK-5 reduced live bacteria and EPS proportionally (Fig. 6E and F). Representative CLSM images of live bacteria and EPS in the 3-day and 3-week biofilms treated with different antimicrobial agents are shown in Fig. 7.

## Discussion

Previous research has primarily focused on *S. mutans* [8, 27], or biofilm models consisting of a limited number of species constructed in a laboratory [28]. In this study, subgingival plaque biofilm from two healthy subjects was employed. The oral biofilm model has been utilized to evaluate the effectiveness of endodontic antimicrobial agents [23, 29]. CLSM is widely recognized as a non-invasive methodology that preserves the biofilm’s architectural integrity while allowing for the visualization and quantification of the three-dimensional architecture of EPS and the extracellular pH of oral biofilms [8, 28]. Therefore, in this study, CLSM along with corresponding fluorescent probes was utilized to investigate the impact of CHX and DJK-5 on the proportion of dead bacteria, in situ pH, and EPS volume within oral biofilms.

In this study, the increasing volume of EPS seemed to have a detrimental effect on the bactericidal effectiveness of both CHX and DJK-5. This was evident as the proportion of dead bacteria decreased over time while the EPS volume increased. The lower layer of the biofilm exhibited lower bacterial death rates from both antimicrobials, which could be attributed to the barrier function of EPS. It has been well-documented that microorganisms within biofilms display heightened resistance to antimicrobials compared to their planktonic counterparts [30]. The primary factor contributing to biofilms resistance against antimicrobial agents is the limited penetration caused by EPS. Moreover, biofilms activate adaptive responses, such as forming long-lasting cells and initiating quorum sensing mechanisms, resulting in physiological heterogeneity within the community [31, 32]. These findings align with previous research highlighting the role of EPS in establishing a protective microenvironment for biofilm microorganisms against the effects of antimicrobials.

The null hypothesis of this study was rejected based on the results, which demonstrated that both DJK-5 and CHX antimicrobial treatments led to a reduction in EPS volume and an increase in the pH of oral biofilms. DJK-5 exhibited greater efficacy than CHX in terms of both bactericidal activity and EPS removal. This was in line with the higher proportion of dead bacteria observed in the DJK-5 group. Notably, DJK-5 consistently removed slightly more EPS than bacteria, except in the upper layer of the 3-day and 1-week biofilms of donor 1, where the live bacteria/EPS ratio was exceptionally large, possibly because DJK-5 almost completely removed the EPS. In contrast, CHX is a cationic bisbiguanide that readily adheres to negatively charged EPS, impeding its diffusion into biofilms. Recent studies have shown that the EPS of *S. mutans* forms densely packed bacterial islets called EPS microcolonies, effectively restricting the access of CHX to these structures and consequently reducing its efficacy [8, 33]. This could elucidate the relatively weaker killing effect of CHX, particularly within the lower layer of biofilms.

A previous study put forth the hypothesis that the reduction in EPS volume following CHX treatment might be attributed to “biofilm contraction” [30]. This concept involves the interaction between the positively charged CHX molecules and the negatively charged EPS matrix, causing the polymeric strands of the EPS matrix to collapse internally. Since DJK-5 is also cationic, it is imperative to conduct further investigations to ascertain whether the decrease in EPS volume observed after DJK-5 treatment was due to EPS removal or the occurrence of “biofilm contraction”.

Xiao et al. [8] and Hwang et al.[33 ] previously demonstrated that the microcolony structure formed by *S. mutans* EPS contains an acidic core located near the bottom layer at the centre of the microcolony. Consistent with these findings, this study also revealed similar results, with the pH in the upper layer being higher than that in the lower layer. The application of antimicrobials led to a rise in the pH of the biofilms, possibly attributed to the disruption of EPS microcolonies and the elimination of acid-producing bacteria. Nevertheless, the two donors exhibited distinct pH trends over time, possibly due to factors such as bacterial species variation, metabolic activities related to carbohydrates, and EPS formation [8]. In this study, in situ pH visualization of the biofilm was accomplished using a fluorescent probe attached to dextran, which can be integrated into the EPS during bacterial biofilm formation. This step is crucial since the pH of the EPS holds a more significant influence on tooth demineralization or remineralization compared to the bacterial intracellular pH [28].

The present study bears noticeable limitations, primarily stemming from the fact that the in vitro cultured biofilm was procured from subgingival sites of healthy volunteers instead of originating directly from root canals. Nonetheless, it is noteworthy that the subgingival plaque’s composition predominantly encompasses gram-positive and gram-negative facultative and anaerobic bacteria. The progression of dental plaque development follows a structured sequence of events, with initial stages characterized by the predominant colonization of tooth surfaces by facultative and aerobic bacteria. As plaque matures, there is a discernible transition in bacterial proportions, with facultative and anaerobic genera gaining prominence [34]. Bacteria implicated in periodontal and endodontic infections, as well as dental caries, can all be traced back to tooth surfaces. The plaque on the tooth surface is easily collected and available in much greater quantities than microorganisms from the root canal. This discrepancy has prompted the utilization of in vitro oral biofilms cultivated from tooth surfaces, rather than root canals, for testing diverse disinfection strategies [12, 13]. To comprehensively address these limitations, further investigation is imperative, focusing on biofilms obtained directly from the root canal system.

## Conclusion

DJK-5 exhibited superior effectiveness in reducing viable bacteria and EPS volume, as well as in raising extracellular pH, as compared to CHX. This distinction was particularly notable in lower-layer and mature biofilms. Hence, DJK-5 holds promise as a potential substitute for CHX in the management of oral biofilms, particularly when dealing with well-established biofilm structures.

## Data Availability

The corresponding author can provide the datasets used and/or analyzed during the current study upon reasonable request.
